# Subtype-specific differences in human immunodeficiency virus type 1 co-receptor usage in Northern Kenya

**DOI:** 10.1186/1742-4690-9-S2-P145

**Published:** 2012-09-13

**Authors:** JM Kingoo, M Muriuki, M Gicheru, Z Ng’ang’a, V Okoth, J Kinyua, N Lagat, ZO Lagat, R Lwembe, SA Khamadi

**Affiliations:** 1 US Military HIV Research Program, KEMRI/WRP CRC KERICHO, Kericho, Kenya; 2 Kenya Medical Research Institute, Nairobi, Kenya; 3 Jomo Kenyatta University of Agriculture and Technology, Nairobi, Kenya

## Background

HIV-1 phenotype variability plays an important role in HIV-1 transmission and pathogenesis of AIDS. The basis of heterogeneity between the phenotypes is known to be the differential use of chemokine receptors as co-receptors for viral entry. Beta Chemokine receptor-5 (CCR5) using variants are associated with acute infections, macrophage tropism, non syncytium inducing (NSI) phenotype and slow progression to AIDS. Alpha chemokine receptor-4 (CXCR4) using variants evolve later in infection in about 50 % of the patients and are associated with T cell lines tropism, syncytium induction, accelerated T cell depletion and Rapid progression to AIDS. HIV-1 co-receptor usage and phenotypes are mapped in the third variable region (V3) of the gp120 env gene.

## Methods

One hundred and thirty five (135) whole blood samples were collected from HIV-1 infected patients in Northern Kenya. Proviral DNA was extracted using DNazol and ethanol precipitation. HIV-1 V3 region was amplified by nested PCR using C2V3 primers. Amplicons were sequenced directly using Big Dye technology in the ABI Prism Genetic Analyzer to generate gp120 V3 loop sequences. Bioinformatics tools were used to determine co-receptor usage from the sequences obtained.

## Results

CXCR4 usage was most dominant with 94 out of 135 samples(69.6 %) using this co-receptor compared to CCR5 which was used by 29 of the 135 (21.8 %) samples and dual co-receptor usage found in 12 of the 135(8.6%)samples tested.

## Conclusion

Most patients were inferred to be in late stages of infection and likely to be rapidly progressing to AIDS. Based on these results from co-receptor usage data, HIV-1 incidence rate was likely to be low. Co-receptor usage was observed to be associated with HIV-1 subtypes. Poor alignment of the Northern Kenya sequences with the training data for the classifier software necessitates the need for development of Bioinformatic software relevant for locally circulating HIV-1 subtypes.

